# Identifying patterns in treatment response profiles in acute bipolar mania: a cluster analysis approach

**DOI:** 10.1186/1471-244X-8-65

**Published:** 2008-07-29

**Authors:** Ilya A Lipkovich, John P Houston, Jonna Ahl

**Affiliations:** 1 Lilly Research Laboratories, Eli Lilly and Company, Indianapolis, IN 46221, USA

## Abstract

**Background:**

Patients with acute mania respond differentially to treatment and, in many cases, fail to obtain or sustain symptom remission. The objective of this exploratory analysis was to characterize response in bipolar disorder by identifying groups of patients with similar manic symptom response profiles.

**Methods:**

Patients (n = 222) were selected from a randomized, double-blind study of treatment with olanzapine or divalproex in bipolar I disorder, manic or mixed episode, with or without psychotic features. Hierarchical clustering based on Ward's distance was used to identify groups of patients based on Young-Mania Rating Scale (YMRS) total scores at each of 5 assessments over 7 weeks. Logistic regression was used to identify baseline predictors for clusters of interest.

**Results:**

Four distinct clusters of patients were identified: Cluster 1 (n = 64): patients did not maintain a response (YMRS total scores ≤ 12); Cluster 2 (n = 92): patients responded rapidly (within less than a week) and response was maintained; Cluster 3 (n = 36): patients responded rapidly but relapsed soon afterwards (YMRS ≥ 15); Cluster 4 (n = 30): patients responded slowly (≥ 2 weeks) and response was maintained. Predictive models using baseline variables found YMRS Item 10 (Appearance), and psychosis to be significant predictors for Clusters 1 and 4 vs. Clusters 2 and 3, but none of the baseline characteristics allowed discriminating between Clusters 1 vs. 4. Experiencing a mixed episode at baseline predicted membership in Clusters 2 and 3 vs. Clusters 1 and 4. Treatment with divalproex, larger number of previous manic episodes, lack of disruptive-aggressive behavior, and more prominent depressive symptoms at baseline were predictors for Cluster 3 vs. 2.

**Conclusion:**

Distinct treatment response profiles can be predicted by clinical features at baseline. The presence of these features as potential risk factors for relapse in patients who have responded to treatment should be considered prior to discharge.

**Trial registration:**

The clinical trial cited in this report has not been registered because it was conducted and completed prior to the inception of clinical trial registries.

## Background

Manic episodes in bipolar disorder requiring hospitalization contribute to substantial personal and economic burden on patients, their families and society [1], which could be alleviated with rapid resolution of acute manic symptoms [2]. Unfortunately, patients with acute mania can respond differentially to treatment due to underlying symptomatic dimensions that vary individually, which can result in failure to obtain or sustain symptom remission [3]. Behavioral subtypes in mania have been described [4] and were found to respond differently to treatment [3] even though the pattern of manic symptom reduction in response to treatment was not found to differ. However, this observation was based on endpoint measures, and any treatment-associated differences in the pattern of symptom change that occurred earlier during treatment may have been missed [3]. What might be of potential utility to the clinician would be to identify distinct time-course patterns of acute treatment response.

The objective of the present study was to identify groups of patients with similar response profiles and to construct predictive models for these groups using baseline data.

The present study is unique in that patients with acute mania were assessed daily during the first week of treatment which allowed the capture of symptom changes that characterized their longer-term response to treatment.

## Methods

### Study design and patient population

This was a post-hoc analysis of patients (n = 222) with bipolar mania or mixed-episode, with or without psychotic features, from a randomized, double-blind clinical trial comparing olanzapine (n = 125; 5–20 mg/d) and divalproex (n = 123; 500–2500 mg/d) in a 3-week acute phase [5] followed by a 44-week maintenance phase [6]. Details of this study have been reported elsewhere, and the following is a brief summary. Acute mania was assessed as having Young-Mania Rating Scale (YMRS) [7] total scores ≥ 20 at baseline. Depressive symptoms were assessed with the Hamilton Rating Scale for Depression (HAMD) [8]. Patients were assessed daily during the first week, weekly for the following 2 weeks, and then bi-weekly for the remainder of the study (a total of 7 weeks for this analysis).

During this study period, remission was defined as having YMRS total scores ≤ 12, and sustained remission was defined as having YMRS total scores ≤ 12 for at least 2 subsequent visits including the last one (discontinuation or at Week 7, whichever occurred first). Relapse (ongoing mania or recurring mania post-baseline after treatment initiation) was defined as having YMRS total scores ≥ 15 at any assessment, and "sustained relapse" (sustained manic episode) was defined as having YMRS total scores ≥ 15 for at least 2 subsequent visits including the last one (discontinuation or at Week 7, whichever occurred first). To facilitate clinical interpretation of the resulting clusters, sustained remission and sustained relapse were evaluated as binary outcomes for each cluster.

### Statistical methods

The objective of the present analysis was to identify distinct response-to-treatment profiles in acute mania that may be representative of certain types of patients, their disease history, baseline conditions, or treatment regimen. An easy-to-implement way of identifying different patterns of treatment response is by clustering estimated regression coefficients fitted to each individual patient's response profile [9-11]. To achieve this, a method of hierarchical clustering based on Ward's distance was applied to the estimated coefficients of orthogonal polynomial regressions of the third degree that were fitted to YMRS total scores during the first 7 weeks of treatment. To smooth YMRS profiles, splines were applied to the data (prior to fitting orthogonal polynomials) using penalized least-squares estimates from a non-parametric regression (SAS^® ^PROC TPSPLINE). This procedure adapts to the pattern of individual profiles by varying model complexity for individual curves. While attempting to reach good fit to the data, this procedure also avoids excessive roughness or rapid variation [12,13]. The outlined procedure of approximating data using orthogonal basis functions (here polynomials) and clustering estimated coefficients appears quite naturally in the context of functional data analysis [9-11,14]. Specifically, when using orthogonal polynomials, the coefficients have the following natural interpretation, independently of other terms included: the coefficient at linear term represents an overall trend in the outcome profile, the coefficient at quadratic term is the rate of change in the expected outcome at time t, and so on [11].

The number of clusters that best represented patient response to treatment as revealed by hierarchical clustering was selected by comparing the percent variation accounted for by the clusters (R^2^) achieved at each level of data aggregation. The level(s) that corresponded to subsequent sharp deterioration of R^2 ^were selected for further inspection, and the clusters that were the most interpretable in terms of clinical relevance were selected for final presentation.

One difficulty encountered with this dataset was the large number of patient dropouts. In order to regularize the data prior to fitting the curves, profiles of patients who discontinued during Weeks 2–7 of treatment were completed using the average of 50 imputed values generated with Bayesian regression fitted to the previous outcomes at every time point past the first week (SAS^® ^PROC MI). This imputation procedure is consistent with the likelihood of observed data and is not expected to introduce any bias when evaluating representative curve shapes, as long as data are missing at random (that is, the probability of dropouts does not depend on unobserved outcomes after the observed outcomes have been accounted for).

To identify baseline variables that may have been contributing to membership in a particular cluster, logistic regression (SAS^® ^PROC LOGISTIC) with stepwise variable selection was used. Baseline variables included in the analysis were: HAMD total scores, individual YMRS items scores, gender, ethnicity, age, weight, body mass index (BMI), index episode and/or illness features (mixed, psychotic, rapid cycling), number of previous manic, mixed, or depressive episodes in the previous year/or lifetime, number of hospitalizations, age at onset of illness, and treatment during the study (olanzapine vs. divalproex).

## Results

Baseline characteristics of patients included in this analysis are summarized in Table 1. Patients were mostly white, in their forties; nearly half were experiencing a mixed-episode and/or had psychotic features. The average number of manic episodes in the previous year was about 4.

**Table 1:** Baseline characteristics of patients who had at least one week of active treatment.

	Olanzapine (n = 111)	Divalproex (n = 111)
YMRS total, mean (SD)	27.3 (5.0)	27.8 (6.7)
HAMD-21 total, mean (SD)	15.1 (7.5)	13.4 (6.9)
Gender (% male)	42.3	41.4
Age, year, mean (SD)	40.15 (12.2)	42.3 (11.8)
Ethnicity (% white)	77.5	84.7
Weight, kg, mean (SD)	80.4 (20.5)	79.2 (20.9)
Mixed episode (%)	47.7	39.6
Psychotic features (%)	49.5	39.6
Rapid cycling (%)	61.3	52.3
Number of manic episodes in previous year, mean (SD)	4.8 (7.3)	3.6 (6.2)

**Figure 1:**
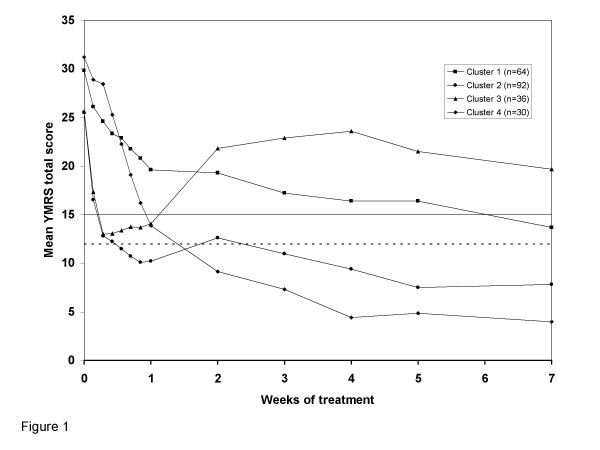
**Response profiles of clusters**. Cluster 1, slow responders who subsequently relapsed. Cluster 2, early symptom improvement in patients who achieved sustained remission. Cluster 3, early symptom improvement in patients who subsequently relapsed. Cluster 4, slow responders who subsequently remitted. The horizontal solid line represents the threshold for relapse (YMRS total score of 15), and the horizontal dashed line represents the threshold for remission (YMRS total score of 12).

Four clusters were identified that best represented patient response to treatment. This identification was based upon comparing the proportion of variation (R^2^) accounted for by joining the 2 closest clusters when trying to reduce the number of clusters that resulted from the analysis. R^2 ^started deteriorating substantially at levels corresponding to solutions with 5 to 4 clusters. The solution with 5 clusters did not introduce substantially different patterns, whereas 4 clusters seemed to capture distinct mania course profiles as shown in Figure 1.

**Figure 2:**
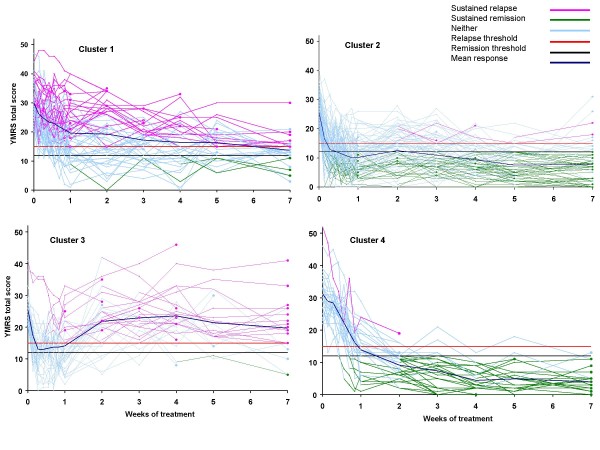
**Clusters with individual patient response profiles**. Individual patient response profiles within each cluster. Colors within each profile were assigned as follows: "magenta" for all time points starting when sustained relapse criterion was first met (YMRS total ≥ 15) until week 7 or discontinuation; "green" for all time points starting when sustained remission criterion was first met (YMRS total ≤ 12) until week 7 or discontinuation; "light blue" for all the time points when neither sustained relapse nor remission criterion were met. Horizontal lines indicate the cut-offs for relapse (red) and remission (black). The dark blue line represents the mean response. A. Cluster 1 patients (n = 64) showing slower response, most of whom had a sustained relapse (magenta lines) or did not meet relapse or remission criteria (blue lines). B. Cluster 2 patients (n = 92) showing rapid early improvement, and most of them had a sustained remission (green lines). C. Cluster 3 patients (n = 36) showing rapid early improvement, then most subsequently relapsed (magenta lines). D. Cluster 4 patients (n = 30) showing slower response, and most of them had a sustained remission (green lines).

In order to provide a visual assessment of the distinctness of the clusters, individual mania profiles (YMRS total scores) of the patients within each cluster were plotted (Figure 2A–D). To facilitate clinical interpretation of the clusters, the individual responses were characterized in terms of 2 binary outcomes: sustained remission and sustained relapse. This was implemented by assigning different colors to illustrate when a patient achieved a particular outcome. More specifically, the color magenta was used to represent when sustained relapse criterion was first met (YMRS total ≥ 15) by the patient, and this color was continued for all subsequent time points until Week 7 or discontinuation. Sustained remission (YMRS total ≤ 12) was represented by the color green starting with when the criterion was first met by the patient, and this color was continued for all subsequent time points until Week 7 or discontinuation. Light blue was used for all time points when neither criterion was met by the patient. Inspecting the multicolored raw curves within their respective clusters provides reassurance that the clusters were not simple artifacts induced by various data transformations (imputation of missing data, smoothing and fitting regressions) and the clustering algorithm itself.

Cluster 1 (Figure 2A) included patients (n = 64) most of whom (95%) did not attain sustained remission of manic symptoms, and who largely (over 50% of them) had a sustained relapse. In this cluster, mania symptoms gradually improved during the first week (mean YMRS total scores = 19.6 [SD 8.8] at Day 7), and 46% of the patients were discharged from the hospital by the end of the first week. In the following weeks, mania symptoms of patients in Cluster 1 improved slightly, but by the end of Week 7 most of these patients still had not remitted indicating poor response to treatment.

Cluster 2 (Figure 2B) included patients (n = 92) who experienced rapid improvement in their symptoms in the first 2 days, and by Day 7, mean YMRS total score was 10.2 (5.8), and 86% were discharged. Most of the patients in this cluster (66%) reached a sustained remission and continued to improve for the duration of the study, and only 5% had a sustained relapse.

Cluster 3 (Figure 2C) included patients (n = 36), who also experience rapid improvement in their symptoms within the first 2 days of treatment, but their symptoms had worsened slightly by Day 7, even so 84% were discharged during the initial week of treatment. Most (82%) of the patients in Cluster 3 had met relapse criterion at Week 2 (n = 34, mean YMRS total score of 21.8 [8.4]), and their YMRS scores did not improve substantially thereafter: 72% of them had maintained relapse until the end of the 7-week period (or discontinuation). There was only one patient (3%) in this cluster who had a sustained remission.

Cluster 4 (Figure 2D) included patients (n = 30) whose mania symptoms improved gradually during the first week and 66% were discharged. Most (83%) of the patients in this cluster were remitted by Week 2, and 80% maintained their remission for the duration of the 7-week period (or until discontinuation). There was only one patient (3%) who had a sustained relapse.

The characteristics of the patients within each cluster prior to initiation of treatment are summarized in Table 2. Patients in Clusters 1 and 4 shared similar clinical characteristics at baseline even though they had different response profiles past Week 1 (Figure 1). Patients in both Clusters 1 and 4 tended to have psychotic features, fewer manic episodes in the preceding year, and over 50% received divalproex; they had also similar baseline HAMD mean total scores, which were slightly higher for patients in Cluster 1 (13.6 [7.7] vs. 11.5 [6.2], p = ns). Membership in Clusters 1 and 4 vs. 2 and 3 was predicted by baseline YMRS Item 10 (Appearance) (adjusted OR = 1.77, 95% CI: 1.24–2.53, p = .002), psychotic features (adjusted OR = 6.54, 95% CI: 3.28–13.08, p < .001), and by not being in a mixed index episode at baseline (adjusted OR = 0.19, CI: 0.10–0.39, p < .001). Membership in Clusters 1 and 4 vs. Clusters 2 and 3 was not predicted by randomization to either treatment. None of the baseline characteristics, however, allowed discriminating between membership in Cluster 1 vs. 4.

**Table 2:** Baseline characteristics of patients associated with each cluster.

	Cluster 1 n = 64	Cluster 2 n = 92	Cluster 3 n = 36	Cluster 4 n = 30
Age, y mean (SD)	42.8 (11.8)	40.8 (11.9)	39.7 (12.0)	40.9 (13.3)
Male, %	46.9	38.0	47.2	36.7
White Ethnicity, %	79.7	82.6	75.0	86.7
Weight, kg mean (SD)	77.8 (19.4)	81.4 (20.7)	82.7 (22.5)	75.6 (20.9)
YMRS total, mean (SD)	29.9 (6.1)	25.5 (4.6)	25.6 (4.7)	31.2 (6.7)
YMRS-09: Disruptive-aggressive behavior, mean (SD)	2.0 (1.6)	2.2 (1.5)	1.7 (1.3)	2.1 (1.5)
YMRS-10: Appearance, mean (SD)^†^	1.3 (1.1)	0.6 (0.9)	0.5 (0.8)	1.3 (1.0)
HAMD-21 total, mean (SD)	13.6 (7.7)	14.6 (7.6)	16.8 (5.5)	11.5 (6.2)
Mixed episode, %	28.1	58.7	58.3	13.3
Psychotic features, %	65.6	26.1	30.6	73.3
Rapid cycling, %	39.1	66.3	63.9	56.7
# manic episodes previous year, mean (SD)	2.9 (5.4)	3.9 (5.6)	8.2 (10.7)	2.9 (4.9)
Randomized to olanzapine, %	46.9	60.9	33.3	43.3

Patients in Clusters 2 and 3 also shared certain clinical characteristics at baseline, but had different outcomes (Figure 1): less than one-third of the patients in these clusters had psychotic symptoms, over 50% were in a mixed episode, and over 60% were rapid cycling. Cluster 3 patients were poor responders with an average of 8 manic episodes in the year preceding study entry, and over two-thirds were randomized to divalproex. Cluster 2 patients, who were responders, had on average 4 manic episodes in the preceding year and over 60% were randomized to olanzapine. Membership in Cluster 3 vs. 2 was significantly predicted by randomization to divalproex (adjusted OR = 5.0, CI: 1.88–13.3, p = .001), experiencing a larger number of manic episodes over the previous 12 months (adjusted OR = 1.11, 95% CI: 1.04–1.18, p = .001), lack of disruptive-aggressive behavior (YMRS Item 9, adjusted OR = 0.57, 85% CI: 0.38–0.85, p = .006) and to some extent by more prominent depressive features at baseline (greater HAMD total score; adjusted OR = 1.10, 95% CI: 1.02–1.18, p = .013).

As we found that patients in Cluster 3 differentiated from those in Cluster 2 by having a substantially larger number of previous manic episodes during the preceding year, one may question whether the study investigators considered the baseline history of manic episodes when making a decision to discharge a patient. From visually inspecting the curves along with associated rates of discharge for each cluster, it seems that the investigators tended to discharge patents with robust improvement during the first week, since Clusters 2 and 3 with rapid early response have the highest proportion of early discharges. To formally evaluate this relationship we fitted logistic regression models with early discharge (yes/no as a response) and YMRS scores during the first 7 days and various baseline and historical characteristics evaluated as potential predictors. The stepwise logistic model revealed that the most important predictors of early discharge were baseline YMRS total score (adjusted odds ratio (OR) = 0.89, p < .001), and change in YMRS from baseline to evaluation at Days 2 (adjusted OR = 0.94, p = .026) and 7 (adjusted OR = 0.93, p = .003). Interestingly, the previous number of manic episodes had a marginally significant but positive effect on early discharge (adjusted OR = 1.08, p = .067), which suggests that while early discharge was driven by mania improvement during the first 7 days, the patient's history of recurrence of mania was not considered in making early discharge decisions. Of course, from our data, we cannot evaluate the possible impact on the outcome, had such information been considered and patients with higher number of manic episodes not been discharged.

## Discussion

In this post-hoc analysis of a double-blind clinical trial [5] in patients with bipolar mania or mixed-episode, we identified 4 clusters with different response profiles that could be grouped into 2 contrasting sets of patterns. The first set involved contrasting patients who rapidly improved during the first week of treatment, some of whom either relapsed (Cluster 3) or continued improving (Cluster 2), and a second set contrasting patients who showed less rapid response during the first week of treatment, one group of which did not gain remission (Cluster 1) and the other group continued to improve to remission (Cluster 4). The presence of psychotic features, not being in a mixed episode and the YMRS Appearance item were the most significant predictors of slower initial improvement as represented by Clusters 1 and 4 vs. Clusters 2 and 3. Patients in Clusters 1 and 4 differed in their rates of rapid cycling at baseline, 39.1% vs. 56.7%. Interestingly Cluster 4, which had the higher rates for rapid cycling, eventually gained remission. For Cluster 3 vs. 2, the larger number of previous manic episodes and randomization to divalproex treatment were significant predictors of relapse following rapid reduction in symptoms. The similarity between clinical courses for Clusters 1 and 4 and for Clusters 2 and 3 during the first week of treatment and their markedly different response profiles is interesting, and may have implications about the importance of clinical observation early in treatment of patients in order to avert potential relapse.

Treatment of manic episodes may be difficult when they are complicated by the presence of other features such as depression, psychosis, and anxiety. Attempts have been made to characterize subtypes of mania that may be associated with these other features and that respond differently to treatment. In a study in untreated patients with acute mania, Swann et al [3] utilized factor analysis of behavioral rating scales scores followed by cluster analysis, which yielded 4 mania subtypes they described as anxious-depressive, psychotic, classic euphoria, and irritable. These subtypes were subsequently found to respond differently to treatment with divalproex or lithium [4]. The anxious-depressed subtype had higher distressed appearance scores and was resistant to treatment, the psychotic and classic euphoria subtypes responded to either lithium or divalproex, and the irritable subtype responded better to divalproex than lithium.

In the current study, we used data from a clinical trial focusing primarily on identifying patterns of response that were based on individual mania profiles during the active treatment and not specifically on clustering patients by pre-treatment mania features. Therefore, the clinical characteristics of the clusters presented herein were not as distinct or as definitive as those for the naturalistic subtypes described by Swann et al [3]. However, the response profiles for the clusters in the present study are supported by previous reports. Cluster 4, which was comprised of more psychotic patients, with more severe mania baseline scores, and less severe depressive scores responded to treatment with either divalproex or olanzapine similar to the psychotic mania subtype described by Swann et al [3]. Patients in Cluster 3, who had more severe depressive symptoms at baseline, relapsed within 2 weeks and remained resistant to treatment similar to the anxious-depressed subtype [3]. In addition, Cluster 3 was characterized by having more prior manic episodes, which has been reported by Welge et al [15] to be a predictor of poor response. Cluster 2, which responded well to treatment with either olanzapine or divalproex, may share features with the classic euphoria subtype of Swann et al [3]. Cluster 1 characteristics and response profiles do not appear to share similarities with any of the naturalistic mania subtypes proposed by Swann et al [3].

## Limitations

This was a post-hoc analysis of a study not designed to assess the complexities of treatment response patterns and warrants further study. Furthermore, replication of this method in larger populations of patients may be needed to validate clusters identified based on this limited data. A further limitation is that this cluster analysis deterministically identified mania profiles ignoring the inherent uncertainty of class membership. For example, some patients may have had profiles on the borderline of two response patterns. Ignoring such uncertainty may result in overstating the odds ratios from the logistic regressions predicting class membership. Additionally, the relationships found between subscores of YMRS and class membership probabilities may be somewhat overstated because the baseline YMRS total score was included as part of the outcome profile creating an overlap. It is important to emphasize that the findings of this study are exploratory and not confirmatory; therefore interpretation of all hypothesis testing should be done with caution and merely indicate possible existing patterns that need to be validated in different datasets, perhaps using more powerful clustering procedures like latent class finite mixture modeling [13,16-18]. This type of modeling would evaluate the profiles representing distinct clusters, as well as predictors of class membership, while properly accounting for class uncertainty within a single estimation step. It requires certain parametric assumptions about the curves and underlying mixture distributions, and the results may be highly sensitive to the initial partitioning of observations into clusters. The approach of the present paper to cluster response profiles was exploratory and data-driven, and is similar to that of Tarpley et al. [10,11]. In addition, our purpose was to search for interesting patterns, including those that were unusual and of relatively low occurrence potentially representing an anomaly, which is more in line with the philosophy of exploratory functional data analysis [14].

In an attempt to validate our exploratory findings, we reanalyzed our data utilizing a fully parametric maximum-likelihood based approach proposed in Jones et al [18] (that is very similar to that of Muthen and Shedden [17]) using the freely available SAS PROC TRAJ software [19] developed by Jones and colleagues. The clustering structure of the data obtained using the latent class mixtures was very similar to our original findings, and the variables identified as significant predictors of class membership were virtually identical to those we identified using simple logistic regression. It is important to point out, however, that the methods used in the present analysis have not been validated in a separate study and may have limitations that have yet to be realized.

## Conclusion

Cluster analysis was useful to identify interesting patterns in treatment response profiles that would be difficult to detect using only pre-specified clinical definitions of relapse or response. Patient history and features of manic episode were important for discriminating between these patterns. In particular, the previous number of manic episodes was found to be a risk factor for relapse in patients who initially responded to treatment and therefore should be considered prior to discharge.

## Competing interests

All authors are employees and/or stockholders of Eli Lilly and Company.

## Authors' contributions

IAL designed, conducted, and interpreted the statistical analyses. JA drafted the manuscript, and all three authors contributed equally to the intellectual content. All authors have given final approval of the version for publication.

## Pre-publication history

The pre-publication history for this paper can be accessed here:


